# Author Correction: Targeted inhibition of STAT/TET1 axis as a therapeutic strategy for acute myeloid leukemia

**DOI:** 10.1038/s41467-018-02947-0

**Published:** 2018-02-09

**Authors:** Xi Jiang, Chao Hu, Kyle Ferchen, Ji Nie, Xiaolong Cui, Chih-Hong Chen, Liting Cheng, Zhixiang Zuo, William Seibel, Chunjiang He, Yixuan Tang, Jennifer R. Skibbe, Mark Wunderlich, William C. Reinhold, Lei Dong, Chao Shen, Stephen Arnovitz, Bryan Ulrich, Jiuwei Lu, Hengyou Weng, Rui Su, Huilin Huang, Yungui Wang, Chenying Li, Xi Qin, James C. Mulloy, Yi Zheng, Jiajie Diao, Jie Jin, Chong Li, Paul P. Liu, Chuan He, Yuan Chen, Jianjun Chen

**Affiliations:** 1 Department of Cancer Biology, University of Cincinnati, Cincinnati, OH 45219 USA; 2 Section of Hematology/Oncology, Department of Medicine, University of Chicago, Chicago, IL 60637 USA; 3 Department of Systems Biology, Beckman Research Institute of City of Hope, Monrovia, CA 91016 USA; 4 Department of Hematology, The First Affiliated Hospital, Zhejiang University, Hangzhou, Zhejiang 310003 China; 5 Department of Chemistry, Department of Biochemistry and Molecular Biology, Institute for Biophysical Dynamics, Howard Hughes Medical Institute, University of Chicago, Chicago, IL 60637 USA; 6 Department of Molecular Medicine, Beckman Research Institute of City of Hope, Duarte, CA 91010 USA; 7 Key Laboratory of Luminescence and Real-time Analytical Chemistry (Ministry of Education), College of Pharmaceutical Sciences, Southwest University, Chongqing, 400715 China; 8 Sun Yat-sen University Cancer Center, State Key Laboratory of Oncology in South China, Collaborative Innovation Center for Cancer Medicine, Guangzhou, 510060 China; 9 Division of Oncology, Cincinnati Children’s Hospital Medical Center, Cincinnati, OH 45229 USA; 10 School of Basic Medical Sciences, Wuhan University, Wuhan, 430071 China; 11 Experimental Hematology and Cancer Biology, Cincinnati Children’s Hospital Medical Center, Cincinnati, OH 45229 USA; 12 Developmental Therapeutics Branch, Center for Cancer Research, NCI, NIH, Bethesda, MD 20892 USA; 13 Genetics and Molecular Biology Branch, National Human Genome Research Institute, NIH, Bethesda, MD 20892 USA

Correction to: *Nature Communications* 10.1038/s41467-017-02290-w; published online: 13 Dec 2017

The original version of this Article contained an error in the spelling of the author James C. Mulloy, which was incorrectly given as James Mulloy. This has now been corrected in both the PDF and HTML versions of the Article.

