# Supplementary material for: Targeted inhibition of STAT/TET1 axis as a therapeutic strategy for acute myeloid leukemia
Source: Nat Commun. 2017 Dec 13;8:2099. doi: 10.1038/s41467-017-02290-w (PMC5727390; doi:10.1038/s41467-017-02290-w)
Supplement: Supplementary file 1 — Supplementary Information [file 41467_2017_2290_MOESM1_ESM.pdf]

Supplementary Table 1. Top 20 candidate chemical compounds that showed positive correlation in drug response and *TET1* level in NCI-60 collection.

Candidate	NSC number	Name	Correlation
1	652026	-	0.59
2	633268	-	0.551
3	600290	4',6,7-Triacetoxyisoflavan	0.538
4	82339	WLN: T56 A BN ENTJ A- DT6NJ	0.535
5	534	ACETOXYPHENANTHRYLMERCURY	0.532
6	624167	-	0.525
7	633272	-	0.525
8	622600	-	0.523
9	628949	-	0.512
10	49512	.alpha.,.alpha.'-Dithiobis(dimethylthio)formamide	0.51
11	622589	-	0.508
12	122291	o-Toluenesulfonyl fluoride, 5-[3-[3-[2-chloro-4-(4,6-diamino-2,2-dimethyl-s-triazin-1(2H)-yl)phenoxy]propyl]ureido]-, monoethanesulfonate (8CI)	0.506
13	635297	-	0.506
14	34757	Melicopidine	0.505
15	625882	-	0.502
16	650738	-	0.501
17	634781	-	0.5
18	370284	6-[(3,4,5-Trimethoxyphenyl)-1-pyrrolidinylmethyl]-1,3-benzodioxol-5-ol	0.499
19	667730	-	0.499
20	311068	Pyrimidine, 4-[(2,4-dinitrophenyl)thio]-	0.498

Supplementary Table 2. Correlation analysis of the responsiveness of cancer cells across the NCI-60 panel to NSC-311068 and NSC-370284.

(a) NCI-60 data for input (transcript expression of *TET1* and drug activity of NSC-311068 and NSC-370284).

Cell lines [a]	TET1	311068	370284
BR:MCF7	1.01	-0.03	-1.03
BR:MDA-MB-231	-1.24	-1.26	-0.22
BR:HS 578T	0.33	-0.26	-
BR:BT-549	0.19	0.02	-
BR:T-47D	0.14	0.22	-0.8
CNS:SF-268	0.75	-0.15	-
CNS:SF-295	-0.68	-1.18	-0.46
CNS:SF-539	1.1	0.03	-
CNS:SNB-19	0.78	-1.54	-0.52
CNS:SNB-75	0.01	-0.02	-0.25
CNS:U251	0.69	-0.89	-0.31
CO:COLO 205	-1.01	-0.06	0.26
CO:HCC-2998	-1.23	-0.83	-1.12
CO:HCT-116	0.49	1.28	-0.17
CO:HCT-15	-1.05	-0.26	0.77
CO:HT29	-1.41	0.4	0.44
CO:KM12	-0.94	-0.15	-0.42
CO:SW-620	1.61	1.87	2.17
LE:CCRF-CEM	1.71	1.66	3.17
LE:HL-60(TB)	0.81	2.62	2.94
LE:K-562	2.71	2.2	1.79
LE:MOLT-4	0.76	0.99	1.94
LE:RPMI-8226	-1.13	-0.09	0.28
LE:SR	-1.36	1.24	-
ME:LOX IMVI	-1.09	0.2	-0.08
ME:MALME-3M	0.56	0.64	1.12
ME:M14	0.87	0.41	-0.89
ME:SK-MEL-2	-0.57	-0.08	-0.54
ME:SK-MEL-28	-0.45	-0.62	-0.81
ME:SK-MEL-5	0.37	-0.34	-0.26
ME:UACC-257	-0.19	-0.18	-0.37

**ME:UACC-62**	0.02	-1.04	-0.2
**ME:MDA-MB-435**	0.69	-0.04	0.05
**ME:MDA-N**	0.3	-	0.04
**LC:A549/ATCC**	-0.36	-0.63	-0.6
**LC:EKVX**	-0.06	-0.01	-0.09
**LC:HOP-62**	-0.41	-0.67	-1.23
**LC:HOP-92**	0.48	0.62	0.86
**LC:NCI-H226**	-1.29	-1.17	-0.36
**LC:NCI-H23**	0.29	-0.22	-0.37
**LC:NCI-H322M**	-0.6	-1.11	-1.34
**LC:NCI-H460**	0.63	-0.31	-
**LC:NCI-H522**	1.96	2.25	2.2
**OV:IGROV1**	1.34	0.08	0.17
**OV:OVCAR-3**	0.91	0.53	-0.38
**OV:OVCAR-4**	0.65	-0.01	-0.32
**OV:OVCAR-5**	-0.44	-0.84	-0.97
**OV:OVCAR-8**	-0.5	-0.4	-0.24
**OV:SK-OV-3**	0.18	-1.37	-1.21
**OV:NCI/ADR-RES**	-0.41	-0.67	-0.35
**PR:PC-3**	0.47	-0.55	-0.34
**PR:DU-145**	-0.34	0.12	-0.51
**RE:786-0**	-0.41	0.07	-0.09
**RE:A498**	-1.11	-1.37	-0.68
**RE:ACHN**	0.01	0.11	-0.17
**RE:CAKI-1**	-0.21	1.48	0.03
**RE:RXF 393**	0.14	-0.54	-
**RE:SN12C**	-1.42	-0.52	0.2
**RE:TK-10**	-0.92	-0.25	-0.59
**RE:UO-31**	-1.08	0.85	-0.11
**Range**	4.13	4.16	4.51
**Minimum**	-1.42	-1.54	-1.34
**Maximum**	2.71	2.62	3.17
**Average**	0.02	0	0
**Standard deviation**	0.91	0.92	0.99
**Data type**	Transcript	Compound	Compound
**Total probes or experiments**	39	2	1
**Total after quality control**	33	2	1

(b) Pearsons correlation[b] between *TET1* expression level and drug responsiveness of NSC-311068 or NSC-370284.

Identifier	TET1	311068	370284
TET1	1	0.5	0.51
311068	**0.5**	1	0.78
370284	**0.51**	**0.78**	1

(c) Pearsons correlation[b] between *TET1* expression level and drug responsiveness of NSC-311068 or NSC-370284 in 32 cell lines with relatively high *TET1* expression level (i.e., *TET1* transcript level > 0; see Supplementary Table 2a).

Identifier	TET1	311068	370284
TET1	1	0.66	0.61
311068	**0.66**	1	0.82
370284	**0.61**	**0.82**	1

(d) Pearsons correlation[b] between *TET1* expression level and drug responsiveness of NSC-311068 or NSC-370284 in 28 cell lines with relatively low *TET1* expression level (i.e., *TET1* transcript level ≤ 0; see Supplementary Table 2a).

Identifier	TET1	311068	370284
TET1	1	0.04	-0.33
311068	**0.04**	1	0.5
370284	**-0.33**	**0.5**	1

Note: [a] Cell lines: The cell line abbreviations are as follows: BR-breast; CNS-central nervous system; CO-colon; LE-leukemia; ME-melanoma; LC-lung cancer; OV-ovarian; PR-prostate; RE -renal.

[b] All the Pearson correlation values shown in red are statistically significant (p<0.05).

Supplementary Table 3. IC50 of the top 20 candidate chemical compounds in MONOMAC-6, THP-1, KOCL-48 and KASUMI-1 cells.

	652026	633268	600290	82339	534	624167	633272	622600	628949	49512
**MMC6**	6.88E+04	5.36E-02	2.21E+04	2.05E+01	5.03E+00	7.35E+04	7.90E-01	7.35E+04	2.53E-04	7.46E-05
**THP-1**	1.09E+00	1.92E-06	8.42E-01	6.09E-01	7.70E-01	N/A	1.31E+00	9.49E-04	N/A	8.30E-03
**KOCL-48**	5.09E+00	5.79E-06	1.36E+00	1.30E+00	1.23E+00	5.85E-01	3.28E-01	1.10E-02	1.63E-05	9.34E-01
**KASUMI1**	3.68E-04	4.83E+06	2.00E-06	2.33E-04	6.08E-01	N/A	N/A	5.79E-06	2.47E+00	1.56E+00
**Average**	1.72E+04	1.21E+06	5.53E+03	5.61E+00	1.91E+00	3.68E+04	8.10E-01	1.84E+04	8.24E-01	6.26E-01

	622589	122291	635297	34757	625882	650738	634781	370284	667730	311068
**MMC6**	1.22E+00	8.93E-01	7.59E+00	8.09E+01	8.28E-01	9.58E+00	N/A	3.85E-04	N/A	6.74E-01
**THP-1**	N/A	N/A	1.07E-02	1.16E-01	2.10E-04	1.30E+00	2.29E+00	3.56E-04	7.10E+00	2.35E-04
**KOCL-48**	5.62E+00	4.76E+00	2.20E+00	8.56E+00	1.46E-01	2.02E+00	N/A	3.82E-02	1.30E+00	7.26E-01
**KASUMI1**	2.46E-05	3.81E-04	4.16E-04	2.38E-04	N/A	7.27E-05	N/A	4.93E-06	4.93E+00	4.20E-06
**Average**	2.28E+00	1.89E+00	2.47E+00	2.24E+01	3.25E-01	3.23E+00	2.30E+00	9.75E-03	4.45E+00	3.50E-01

Supplementary Table 4. Genes with recurrent mutations in THP-1 NSC-370284-resistant clones.

Gene	ExonicFunc	AAChange	Chr	Pos	Ref	Obs
MSH3	nonsynonymous SNV	NM_002439:c.G1798A:p.V600I	chr5	80057399	G	A
COG3	nonsynonymous SNV	NM_031431:c.A1903G:p.I635V	chr13	46090371	A	G
IPO5	nonsynonymous SNV	NM_002271:c.G2524A:p.V842I	chr13	98668012	G	A
TMED5	nonsynonymous SNV	NM_001167830:c.T415C:p.W139R	chr1	93621913	A	G
HSP90AB1	nonsynonymous SNV	NM_001271969:c.T593G:p.V198G	chr6	44217836	T	G
CCDC132	nonsynonymous SNV	NM_017667:c.A1877T:p.N626I	chr7	92952944	A	T
MIER2	nonsynonymous SNV	NM_017550:c.G1396A:p.A466T	chr19	307339	C	T
SYF2	nonsynonymous SNV	NM_207170:c.G206C:p.W69S	chr1	25554653	C	G
VDAC2	nonsynonymous SNV	NM_001184823:c.G826C:p.G276R	chr10	76990688	G	C
SWAP70	nonsynonymous SNV	NM_015055:c.A1527T:p.E509D	chr11	9769576	A	T
NOTCH2	frameshift deletion	NM_001200001:c.17_18del:p.6_6del	chr1	120612003	GG	-
MARCKS	nonsynonymous SNV	NM_002356:c.G130C:p.D44H	chr6	114180886	G	C
SRRM2	frameshift deletion	NM_016333:c.5444_5445del:p.1815_1815del	chr16	2815973	GG	-
JAK1	nonsynonymous SNV	NM_002227:c.A893G:p.E298G	chr1	65332646	T	C

Supplementary Table 5. Top 5 signaling pathways and associated diseases of the genes with recurrent mutations in THP-1 NSC-370284-resistant clones.

(**a**) Top 5 canonical pathways involving the genes with recurrent mutations in THP-1 NSC-370284-resistant clones.

Name of Canonical Pathways	*P* value
IL-22 Signaling	1.17E-04
Role of JAK family kinases in IL-6-type Cytokine Signaling	1.27E-04
IL-9 Signaling	2.37E-04
Oncostatin M Signaling	2.37E-04
Role of JAK2 in Hormone-like Cytokine Signaling	2.37E-04

(**b**) Top 5 diseases and disorders involving the genes with recurrent mutations in THP-1 NSC-370284-resistant clones.

Diseases and Disorders	*P* value range
Cancer	4.84E-02 ~ 2.14E-05
Hematological Disease	4.26E-02 ~ 2.14E-05
Immunological Disease	4.26E-02 ~ 2.14E-05
Organismal Injury and Abnormalities	4.84E-02 ~ 2.14E-05
Developmental Disorder	4.13E-02 ~ 2.14E-05

Supplementary Table 6. 30 structural analogs of NSC-370284 from the University of Cincinnati Compound Library.

Structures	Name	MW Formula	Smiles
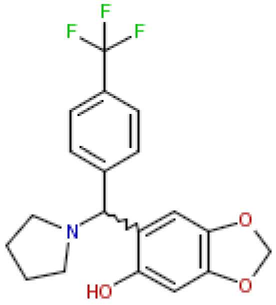	765626	365.3463	c1(cc2c(OCO2)cc1O)C(c(cc3)ccc3C(F)(F)F)N4CCCC4
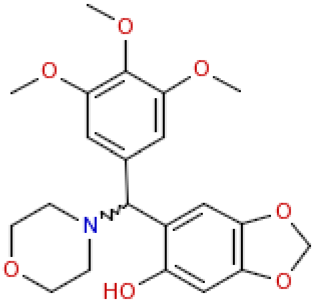	517451	403.4257	O1COc2c1cc(O)c(C(c3cc(OC)c(OC)c(OC)c3)N4CCOCC4)c2
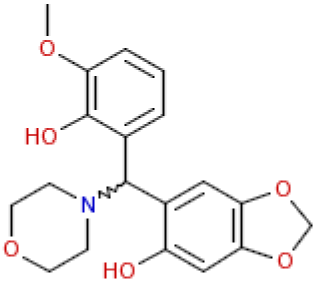	472619	359.3731	O1COc2c1cc(O)c(C(c3c(O)c(OC)ccc3)N4CCOCC4)c2

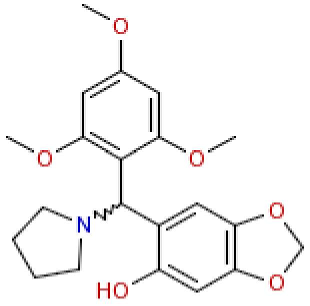	472616	387.4263	O1COc2c1cc(O)c(C(c3c(OC)cc(OC)cc3OC)N4CCCC4)c2
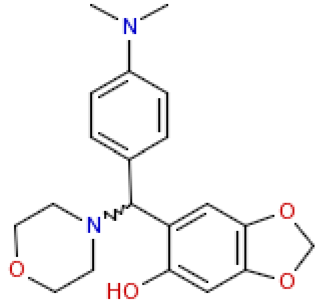	517463	356.4156	O1COc2c1cc(O)c(C(c3ccc(N(C)C)cc3)N4CCOCC4)c2
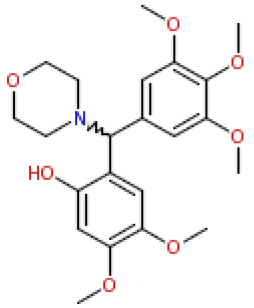	517447	419.4682	N1(C(c2cc(OC)c(OC)c(OC)c2)c(c(O)cc3OC)cc3OC)CCOCC1
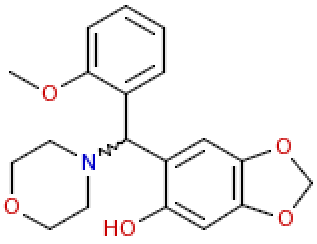	472585	343.3737	O1COc2c1cc(O)c(C(c3c(OC)cccc3)N4CCOCC4)c2

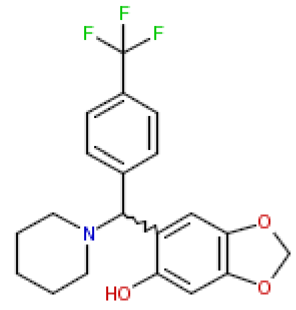	943568	379.3729	c1(cc2c(OCO2)cc1O)C(c(cc3)ccc3C(F)(F)F)N4CCCCC4
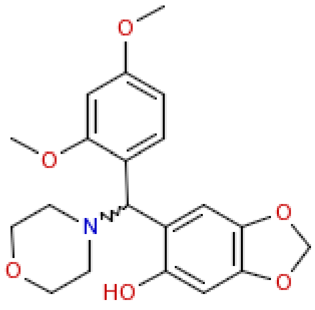	472591	373.3997	O1COc2c1cc(O)c(C(c3c(OC)cc(OC)cc3)N4CCOCC4)c2
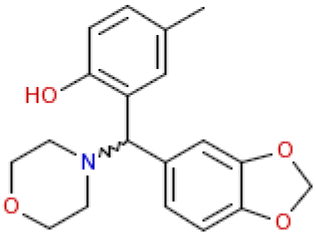	514329	327.3743	O1COc(c1cc2)cc2C(c(cc(C)c3)c(O)c3)N4CCOCC4
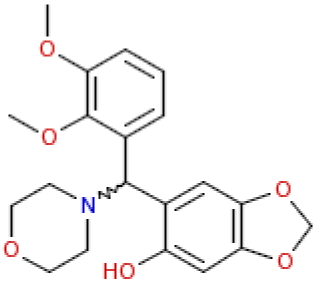	472587	373.3997	O1COc2c1cc(O)c(C(c3c(OC)c(OC)ccc3)N4CCOCC4)c2

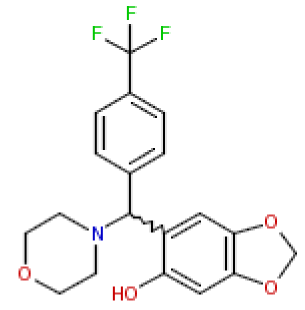	943336	381.3457	c1(cc2c(OCO2)cc1O)C(c(cc3)ccc3C(F)(F)F)N(CCO4)CC4
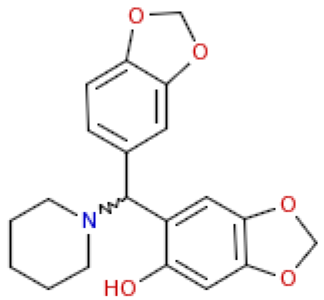	514341	355.3844	O1COc2c1cc(O)c(C(c3cc(c4cc3)OCO4)N5CCCCC5)c2
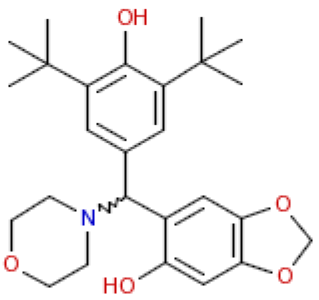	514321	441.5598	O1COc2c1cc(O)c(C(c3cc(C(C)(C)C)c(O)c(C(C)(C)C)c3)N4CCOCC4)c2
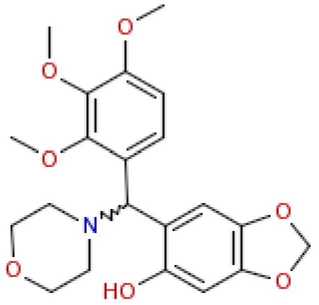	472606	403.4257	O1COc2c1cc(O)c(C(c3c(OC)c(OC)c(OC)cc3)N4CCOCC4)c2

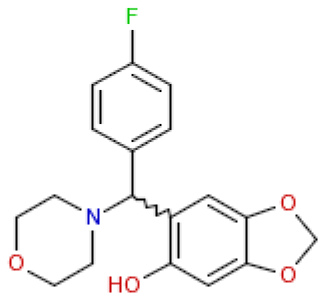	943337	331.3382	c1(cc2c(OCO2)cc1O)C(c3ccc(F)cc3)N(CC4)CCO4
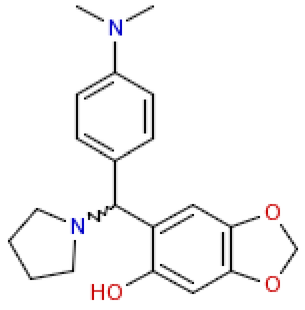	514318	340.4162	O1COc2c1cc(O)c(C(c3ccc(N(C)C)cc3)N4CCCC4)c2
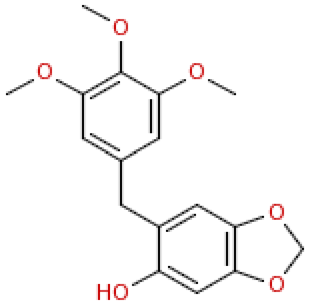	514328	318.3212	O1COc2c1cc(O)c(Cc3cc(OC)c(OC)c(OC)c3)c2
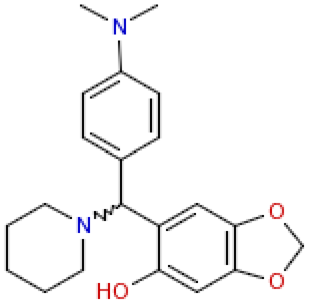	491129	354.4427	O1COc2c1cc(O)c(C(c3ccc(N(C)C)cc3)N4CCCCC4)c2

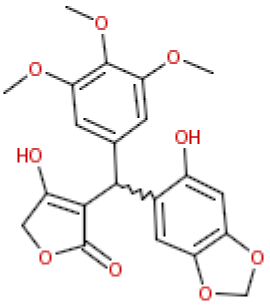	514315	416.3781	O1COc2c1cc(c(C(C(C(O3)=O)=C(O)C3)c4cc(OC)c(OC)c(OC)c4)c2)O
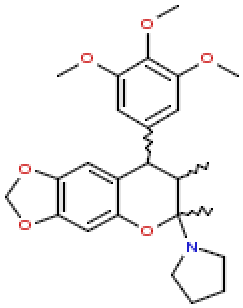	517461	441.5167	O1COc2c1cc3c(C(c4cc(OC)c(OC)c(OC)c4)C(C)C(C)(N5CCCC5)O3)c2
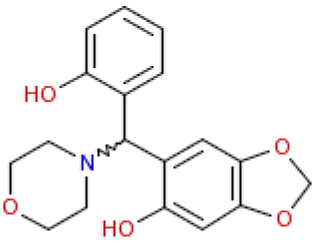	472589	329.3472	O1COc2c1cc(O)c(C(c3c(O)cccc3)N4CCOCC4)c2
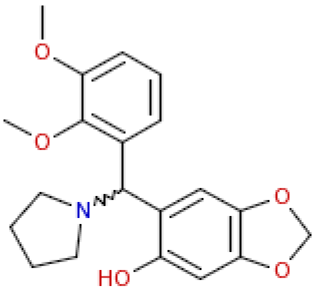	472610	357.4003	O1COc2c1cc(O)c(C(c3cccc(OC)c3OC)N4CCCC4)c2

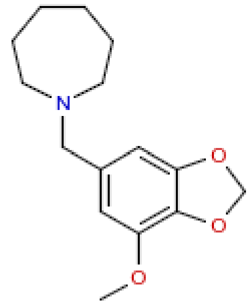	378752	263.3321	c12c(cc(CN3CCCCCC3)cc1OC)OCO2
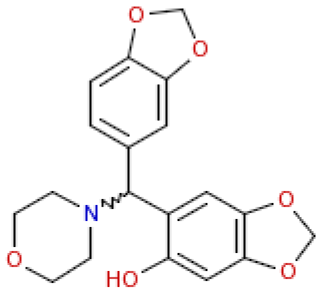	514363	357.3573	O1COc2c1cc(O)c(C(c3cc(c4cc3)OCO4)N5CCOCC5)c2
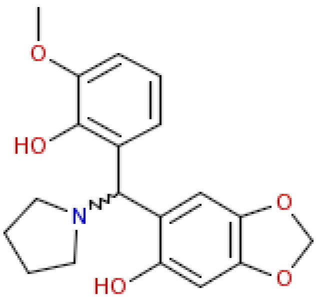	472614	343.3737	O1COc2c1cc(O)c(C(c3cccc(OC)c3O)N4CCCC4)c2
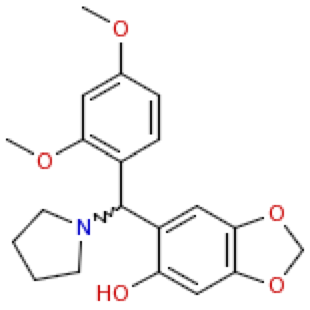	472607	357.4003	O1COc2c1cc(O)c(C(c3ccc(OC)cc3OC)N4CCCC4)c2

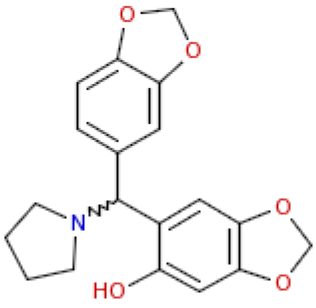	517453	341.3579	O1COc2c1cc(O)c(C(c3cc(c4cc3)OCO4)N5CCCC5)c2
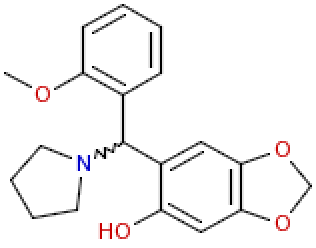	472617	327.3743	O1COc2c1cc(O)c(C(c3ccccc3OC)N4CCCC4)c2
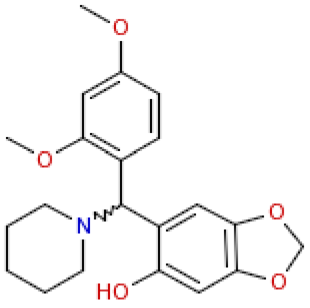	472595	371.4269	O1COc2c1cc(O)c(C(c3ccc(OC)cc3OC)N4CCCCC4)c2

Supplementary Table 7. Acute or long-term effects of NSC-370284 and UC-514321 on mouse blood cell differentiation after 10 consecutive days' administration.

(**a**) Acute effects (24 hrs post the last administration) of NSC-370284 and UC-514321 on mouse blood cell differentiation.

	**WBC (K/µl)**	**NE (K/µl)**	**LY (K/µl)**	**MO (K/µl)**	**EO (K/µl)**	**BA (K/µl)**	**RBC (M/µl)**	**PLT (K/µl)**
**DMSO**	10.32 ± 3.2	1.97 ± 0.65	7.49 ± 2.6	0.27 ± 0.02	0.2 ± 0.08	0.07 ± 0.03	8.61 ± 1.86	552.32 ± 131.22
**370284**	8.35 ± 2.34	1.67 ± 0.21	6.74 ± 2.55	0.22 ± 0.14	0.15 ± 0.01	0.05 ± 0.05	8.55 ± 3.34	460.21 ± 42.42
**514321**	8.64 ± 3.68	1.94 ± 0.56	6.54 ± 2.7	0.27 ± 0.1	0.14 ± 0.12	0.07 ± 0.05	9.54 ± 0.74	559.43 ± 116.9
**Normal range**	1.8-10.7	0.1-2.4	0.9-9.3	0.0-0.4	0.0-0.2	0.0-0.2	6.36-9.42	592-2972

(**b**) Long-term effects (200 days post the last administration) of NSC-370284 and UC-514321 on mouse blood cell differentiation.

	**WBC (K/µl)**	**NE (K/µl)**	**LY (K/µl)**	**MO (K/µl)**	**EO (K/µl)**	**BA (K/µl)**	**RBC (M/µl)**	**PLT (K/µl)**
**DMSO**	11.97 ± 3.26	1.83 ± 0.51	9.79 ± 2.85	0.22 ± 0.09	0.06 ± 0.04	0.01 ± 0.01	8.5 ± 1.59	954.95 ± 99.25
**370284**	10.34 ± 2.19	2.26 ± 0.54	7.68 ± 1.55	0.31 ± 0.03	0.11 ± 0.01	0.03 ± 0.02	7.38 ± 1.7	929.62 ± 154.61
**514321**	11.89 ± 1.64	2.11 ± 0.44	8.55 ± 1.18	0.25 ± 0.05	0.1 ± 0.01	0.07 ± 0.04	7.07 ± 0.42	681.56 ± 402.64
**Normal range**	1.8-10.7	0.1-2.4	0.9-9.3	0.0-0.4	0.0-0.2	0.0-0.2	6.36-9.42	592-2972

Note: Normal C57BL/6 mice were treated with PBS (control), 2.5 mg/kg NSC-370284 or UC-514321, *i.p.*, once every day for 10 days. Shown are data collected at the indicated time points (i.e. 24 hrs (**a**) or 200 days (**b**)) post the last *i.p.* injection of the chemical compounds or DMSO control (DMSO). WBC = white blood cells; NE = neutrophils; LY = lymphocytes; MO = monocytes; EO = eosinophils; BA = basophils; RBC = red blood cells; PLT = platelets. Means ± Standard deviations are shown.

Supplementary Table 8. Maximum tolerated dose (MTD) and median lethal dose (LD_{50}) of NSC-370284 and UC-514321.

**NSC-370284 (LD_{50} = 123.1 mg/kg)**				
**Animals**	**Routs**	**Dose**	**Number of Death**	**Number of Exposure**
C57BL/6	I.P.	200 mg/kg	10	10
	I.P.	150 mg/kg	6	10
	I.P.	113.5 mg/kg	3	10
	I.P.	85.6 mg/kg	1	10
	I.P.	65 mg/kg	0	10
**UC-514321 (LD_{50} = 123.7 mg/kg)**				
**Animals**	**Routs**	**Dose**	**Number of Death**	**Number of Exposure**
C57BL/6	I.P.	200 mg/kg	10	10
	I.P.	150 mg/kg	7	10
	I.P.	113.5 mg/kg	2	10
	I.P.	85.6 mg/kg	1	10
	I.P.	65 mg/kg	0	10

Supplementary Table 9. Blood pharmacokinetic parameters of UC-514321 (data provided as mean ± SD).

**Pharmacokinetic Parameters**	C57BL/6 Mice
	I.P. 15 mg/kg
**Half-life (h)**	11.02±2.51
**AUC $_{0-\infty}$ (μg·h·ml^{-1})**	29.35±2.75
**Mean Residence Time (h)**	16.12±1.79
**Volume of Distribution (L·kg^{-1})**	21.66±1.74
**Clearance (L·kg^{-1}·h^{-1})**	1.36±0.08

Supplementary Table 10. GSEA of NSC-370284-treated or resistant THP-1 cells.

(**a**) Gene clusters enriched in NSC-370284-resistant THP-1 clones as compared with parental THP-1 cells.

NAME	NOM p-val	FDR q-val	FWER p-val
HALLMARK_PROTEIN_SECRETION	0.00	0.00	0.00
HALLMARK_G2M_CHECKPOINT	0.00	0.00	0.00
HALLMARK_MYC_TARGETS_V1	0.00	0.00	0.00
HALLMARK_E2F_TARGETS	0.00	0.00	0.00
HALLMARK_ANDROGEN_RESPONSE	0.00	0.00	0.01
HALLMARK_MTORC1_SIGNALING	0.00	0.01	0.02
HALLMARK_TGF_BETA_SIGNALING	0.01	0.01	0.03
HALLMARK_UV_RESPONSE_DN	0.00	0.02	0.06
HALLMARK_TNFA_SIGNALING_VIA_NFKB	0.00	0.02	0.08
HALLMARK_MITOTIC_SPINDLE	0.00	0.03	0.12
HALLMARK_PI3K_AKT_MTOR_SIGNALING	0.02	0.05	0.19
HALLMARK_APOPTOSIS	0.04	0.15	0.51
HALLMARK_SPERMATOGENESIS	0.03	0.14	0.53
HALLMARK_HYPOXIA	0.02	0.14	0.54
HALLMARK_KRAS_SIGNALING_UP	0.04	0.15	0.59
HALLMARK_OXIDATIVE_PHOSPHORYLATION	0.07	0.25	0.79
HALLMARK_ADIPOGENESIS	0.08	0.24	0.81
HALLMARK_FATTY_ACID_METABOLISM	0.13	0.30	0.89
HALLMARK_IL2_STAT5_SIGNALING	0.13	0.30	0.90
HALLMARK_PANCREAS_BETA_CELLS	0.26	0.34	0.93
HALLMARK_HEME_METABOLISM	0.19	0.34	0.94
HALLMARK_UV_RESPONSE_UP	0.24	0.37	0.96
HALLMARK_INFLAMMATORY_RESPONSE	0.26	0.46	0.98
HALLMARK_UNFOLDED_PROTEIN_RESPONSE	0.34	0.48	0.99
HALLMARK_ALLOGRAFT_REJECTION	0.35	0.49	1.00
HALLMARK_COMPLEMENT	0.35	0.48	1.00
HALLMARK_GLYCOLYSIS	0.35	0.50	1.00
HALLMARK_INTERFERON_GAMMA_RESPONSE	0.50	0.58	1.00
HALLMARK_P53_PATHWAY	0.62	0.70	1.00
HALLMARK_PEROXISOME	0.63	0.75	1.00
HALLMARK_IL6_JAK_STAT3_SIGNALING	0.76	0.87	1.00
HALLMARK_CHOLESTEROL_HOMEOSTASIS	0.79	0.89	1.00
HALLMARK_INTERFERON_ALPHA_RESPONSE	0.87	0.93	1.00
HALLMARK_ANGIOGENESIS	0.91	0.98	1.00

(**b**) Gene clusters enriched in DMSO (Ctrl) treated THP-1 cells as compared with NSC-370284-treated cells.

NAME	NOM p-val	FDR q-val	FWER p-val
HALLMARK_E2F_TARGETS	0.00	0.00	0.00
HALLMARK_G2M_CHECKPOINT	0.00	0.00	0.00
HALLMARK_GLYCOLYSIS	0.00	0.06	0.05
HALLMARK_MYC_TARGETS_V1	0.00	0.08	0.08
HALLMARK_DNA_REPAIR	0.85	1.00	0.78
HALLMARK_MITOTIC_SPINDLE	1.00	1.00	0.85
HALLMARK_SPERMATOGENESIS	0.98	0.96	0.86

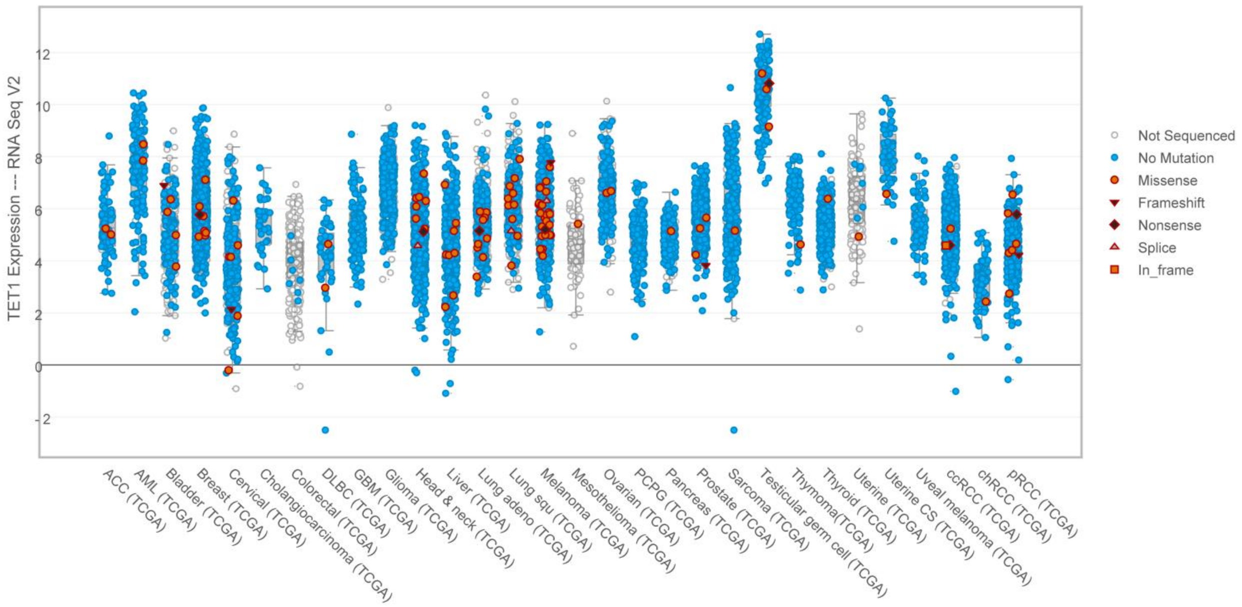

Supplementary Figure 1. Expression pattern of *TET1* in TCGA database. *TET1* gene expression levels were analyzed through cBioPortal website.

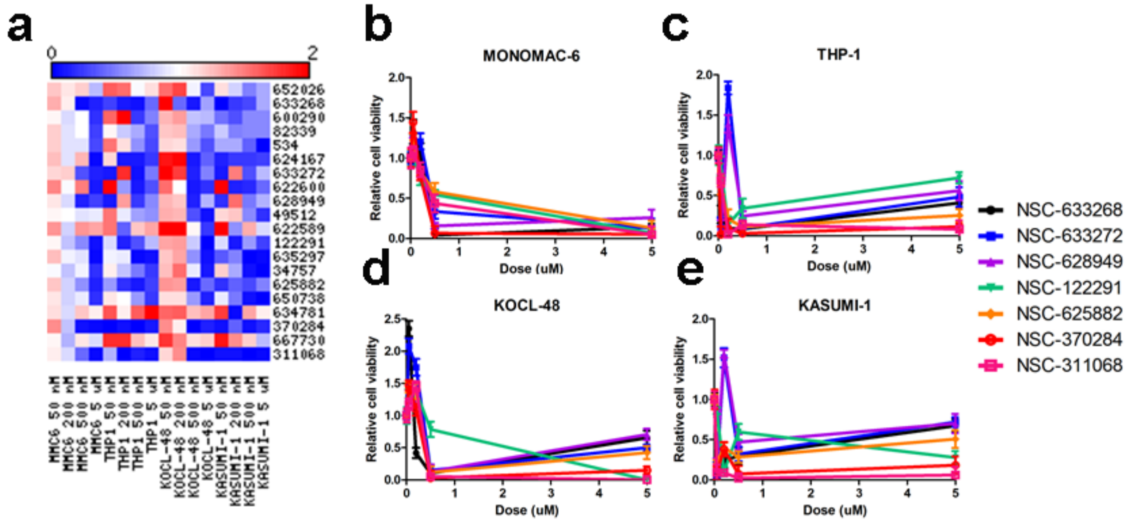

Supplementary Figure 2. Cell viability screening of the top 20 chemical compounds that showed positive correlation between drug response and *TET1* level in NCI-60 panel collection. MONOMAC-6 (MMC6), THP-1, KOCL-48 and KASUMI-1 cells were treated with the 20 top candidate chemical compounds individually at indicated doses for 48 hrs. Heatmap (**a**) and dose curves (**b-e**) are shown.

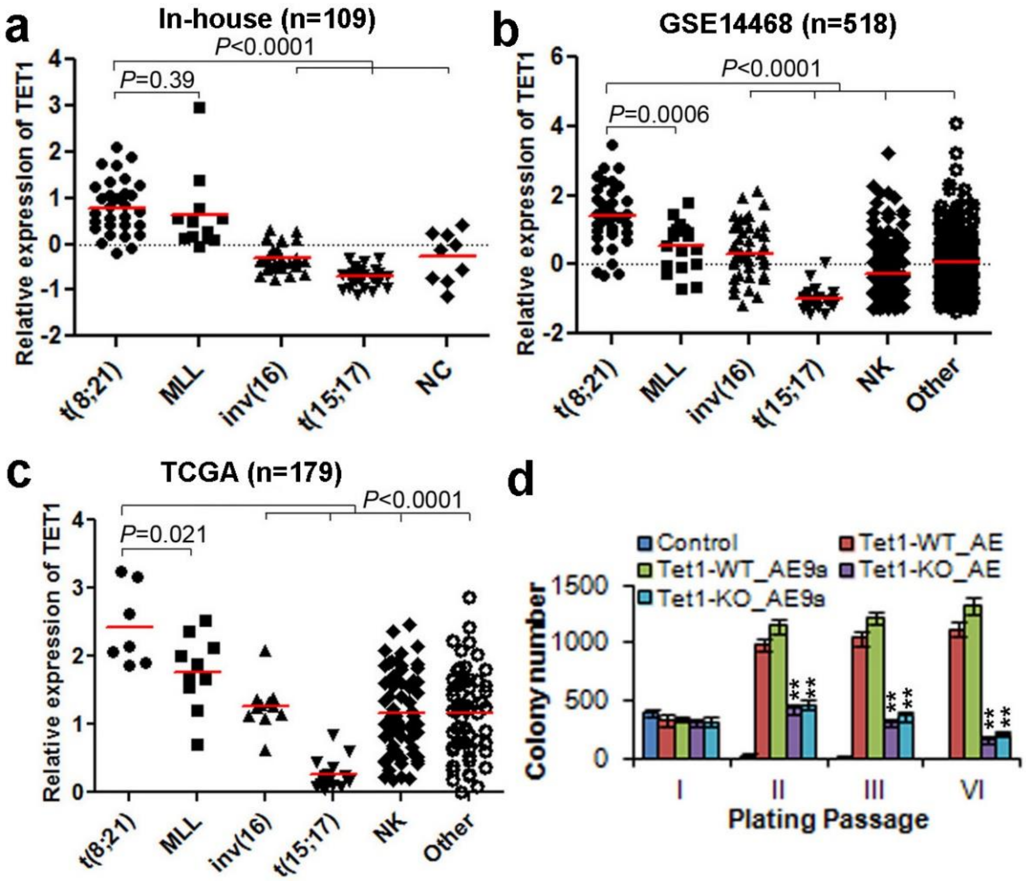

Supplementary Figure 3. High expression and oncogenic role of *TET1* in t(8;21) AML. (**a-c**) Comparison of *TET1* expression between t(8;21) AML and other subtypes of AML or normal control (NC) samples. (**a**) In our In-house 109-sample dataset, including 30 t(8;21), 12 *MLL*-rearranged (MLL), 27 inv(16), and 31 t(15;17) AML, along with 9 normal control (NC) samples. (**b**) In the Netherlands 518-sample dataset (GSE14468), including 38 t(8;21), 19 *MLL*-rearranged, 42 inv(16), 25 t(15;17), 214 normal karyotype (NK) and 180 other AML samples. (**c**) In the TCGA 179-sample dataset, including 7 t(8;21), 11 *MLL*-rearranged, 11 inv(16), 16 t(15;17), 75 normal karyotype (NK) and 59 other AML samples. Two-tailed *t*-test was used to calculate the *P* values. Bars show the median values. (**d**) *Tet1* knockout inhibits t(8;21) fusion-induced cell transformation. Colony-forming/ replating assays of wild-type or $Tet1^{-/-}$ mouse BM progenitor cells transduced with MSCV-PIG-*AE* (AE), or MSCV-PIG-*AE9a* (AE9a). Wild-type cells transduced with MSCV-PIG serve as a negative control. **, *P*<0.01, two-tailed *t*-test.

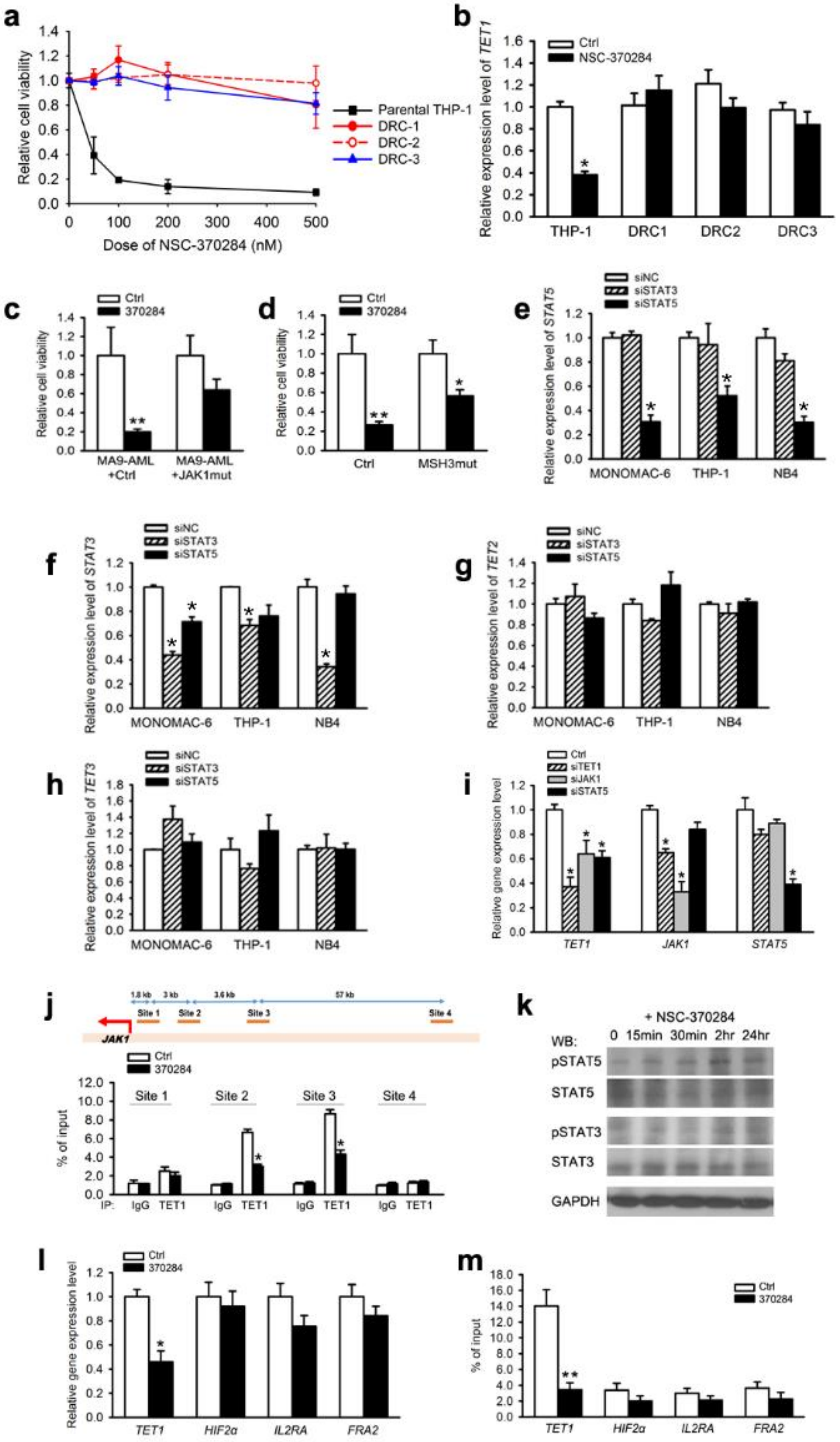
a
Relative cell viability
Dose of NSC-370284 (nM)
Parental THP-1
DRC-1
DRC-2
DRC-3
b
Relative expression level of TET1
Ctrl
NSC-370284
THP-1
DRC1
DRC2
DRC3
c
Relative cell viability
Ctrl
370284
MA9-AML +Ctrl
MA9-AML +JAK1mut
d
Relative cell viability
Ctrl
370284
Ctrl
MSH3mut
e
Relative expression level of STAT5
siNC
siSTAT3
siSTAT5
MONOMAC-6
THP-1
NB4
f
Relative expression level of STAT3
siNC
siSTAT3
siSTAT5
MONOMAC-6
THP-1
NB4
g
Relative expression level of TET2
siNC
siSTAT3
siSTAT5
MONOMAC-6
THP-1
NB4
h
Relative expression level of TET3
siNC
siSTAT3
siSTAT5
MONOMAC-6
THP-1
NB4
i
Relative gene expression level
Ctrl
siTET1
siJAK1
siSTAT5
TET1
JAK1
STAT5
j
1.8 kb
3 kb
3.6 kb
57 kb
Site 1
Site 2
Site 3
Site 4
JAK1
Ctrl
370284
% of input
IP:
IgG
TET1
k
+ NSC-370284
WB:
0
15min
30min
2hr
24hr
pSTAT5
STAT5
pSTAT3
STAT3
GAPDH
l
Relative gene expression level
Ctrl
370284
TET1
HIF2α
IL2RA
FRA2
m
% of input
Ctrl
370284
TET1
HIF2α
IL2RA
FRA2

Supplementary Figure 4. NSC-370284 regulates *TET1* expression through targeting STAT3/5. (**a**) THP-1 parental cells or representative drug-resistant clones (i.e., DRC-1, 2, 3) were treated with NSC-370284 at indicated doses for 48 hrs. Relative cell viabilities are shown. (**b**) THP-1 parental cells or drug resistant clones were treated with DMSO control or 500 nM NSC-370284 for 48 hrs. Levels of *TET1* transcripts are shown. (**c**) Relative viability of *MLL-AF9*-AML cells transduced with MSCV-PIG-$JAK1^{A839G}$ mutant (Mut) or MSCV-PIG vector (ctrl) and then treated with DMSO or NSC-370284 (50 nM) for 48 hours. (**d**) Relative viability of THP-1 cells transduced with pLJM1-$MSH3^{V600I}$ or pLJM1-EGFP control and then treated with DMSO or NSC-370284 (50 nM) for 48 hours. (**e-h**) MONOMAC-6, THP-1 and NB4 cells were transfected with control siRNA (siNC), si*STAT3*, or si*STAT5*. Expression levels of *STAT5* (**e**), *STAT3* (**f**), *TET2* (**g**) and *TET3* (**h**) were detected by qPCR 48 hrs post-transfection. (**i**) THP-1 cells were transfected with control siRNA (siNC), si*TET1*, si*JAK1* or si*STAT5*. Gene expression levels were detected by qPCR 48 hrs post-transfection. MONOMAC-6 cells were treated with DMSO control or 500 nM NSC-370284. (**j**) ChIP-qPCR assay was carried out 48 hrs after drug treatment. Enrichment of TET1 or IgG at the *JAK1* promoter region and other regions are shown. (**k**) MONOMAC-6 cells were treated with 250 nM NSC-370284 for 0 min, 15 min, 30 min, 2 hrs, and 24 hrs. Protein levels were detected thereafter. (**l,m**) THP-1 cells were treated with DMSO or 25 nM NSC-370284 for 24 hrs. Expression levels of *TET1, HIF2α, IL2RA and FRA2* (**l**) were detected through qPCR. The enrichment of STAT5 on the promoters of *TET1* (Site 2; CpG), *HIF2α*[1], *IL2RA*[2] *and FRA2*[3] (**m**) was determined through ChIP-qPCR assays. *, $P<0.05$; **, $P<0.01$, two-tailed *t*-test. Error bar indicates SD of triplicate experiments.

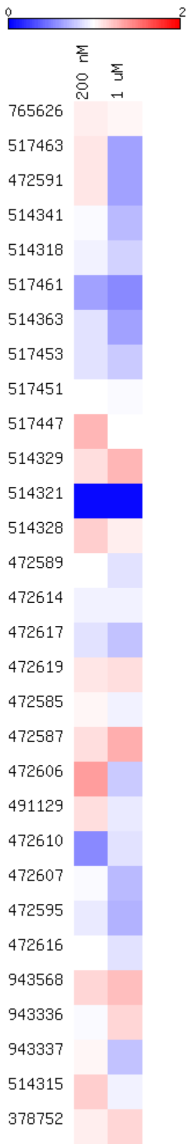

Supplementary Figure 5. Effects of the 30 analogues of NSC-370284 on the viability of MONOMAC-6 cells. MONOMAC-6 cells were treated with the 30 candidate analogues individually at indicated doses for 48 hrs. Heatmap of relative cell viability (as compared with DMSO treated control group) is shown.

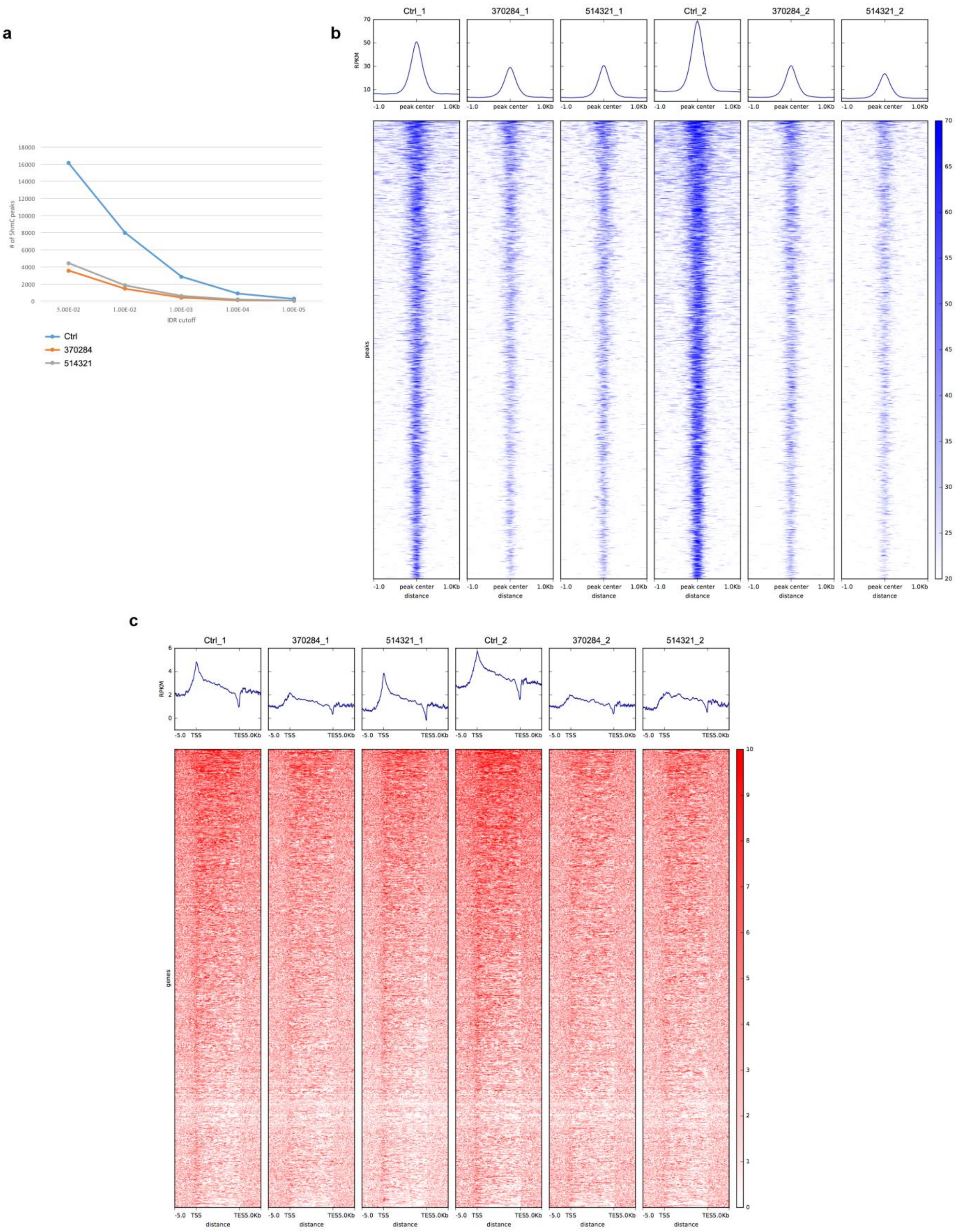

Supplementary Figure 6. Comparison of 5hmC enrichment between NSC-370284 or UC-514321-treated AML cells and control (DMSO-treated) cells. (**a**) Comparison of 5hmC peak

numbers across the whole genome in ML-2 AML cells treated with NSC-370284 or UC-514321, or DMSO (control). 5hmC peak counts at different IDR cutoffs were shown. (**b**) Read density across 5hmC enriched regions. A unified catalog of 5hmC enriched regions was generated (see Methods). Shown are heatmaps rank-ordered by reads per kilobase of exon model per million mapped reads (RPKM) of 5hmC-Seal samples minus that of the input samples. (**c**) Read density across all RefSeq genes. Shown are heatmaps of 5hmC read density changes across all RefSeq gene loci and 5 kb flanking regions (minus input values).

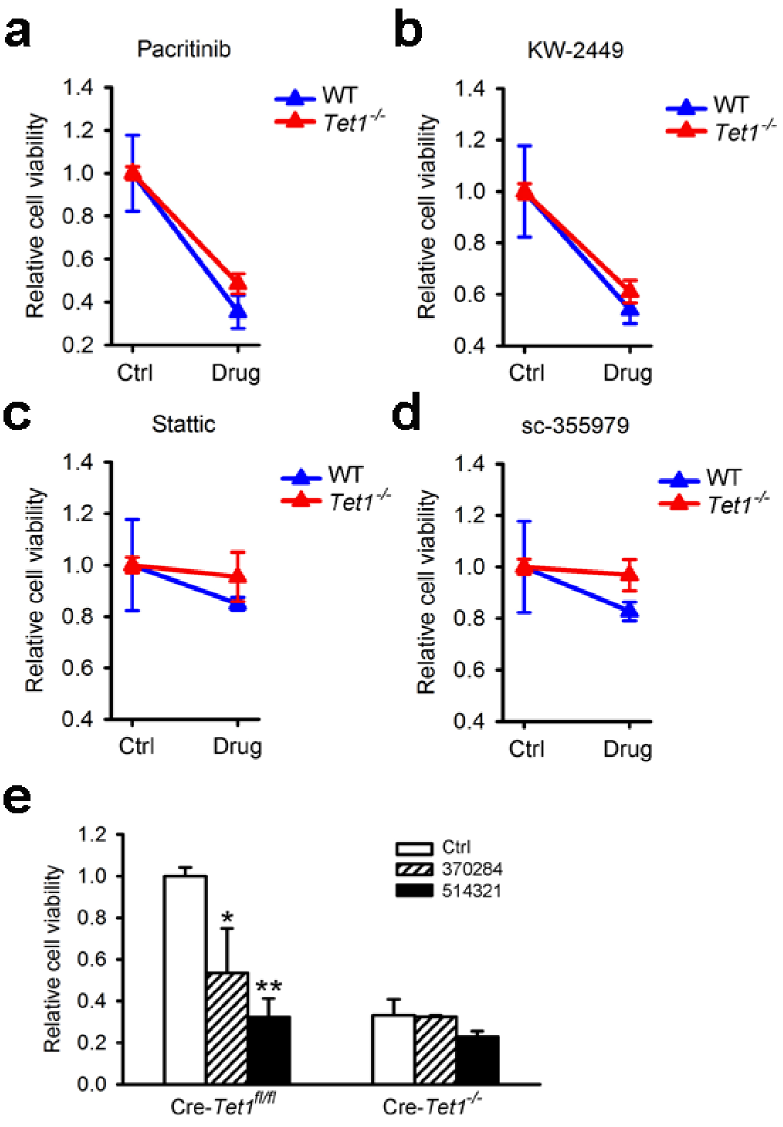

Supplementary Figure 7. Unlike those of NSC-370284 and UC-514321, the effects of a set of JAK/STAT inhibitors are less TET1-dependent. (**a-d**) BM progenitor cells of wild-type or *Tet1*$^{-/-}$ mice were retrovirally transduced with *MLL-AF9*. Infected cells were treated with 200 nM JAK/STAT inhibitors individually, including Pacritinib (**a**), KW-2449 (**b**), Stattic (**c**) or sc-355979 (**d**), for 48 hrs. Relative cell viabilities are shown. (**e**) Cre-*Tet1*$^{fl/fl}$ mouse BM progenitor cells were retrovirally transduced with *MLL-AF9*. Transduced cells were induced with polyI:C for 7 days, and then treated with 500 nM NSC-370284, UC-514321, or DMSO control for 48 hrs. Cell viabilities (normalized to non-induced Cre-*Tet1*$^{fl/fl}$ treated with DMSO control) are shown. *, *P*<0.05; **, *P*<0.01, two-tailed *t*-test. Error bar indicates SD of triplicate experiments.

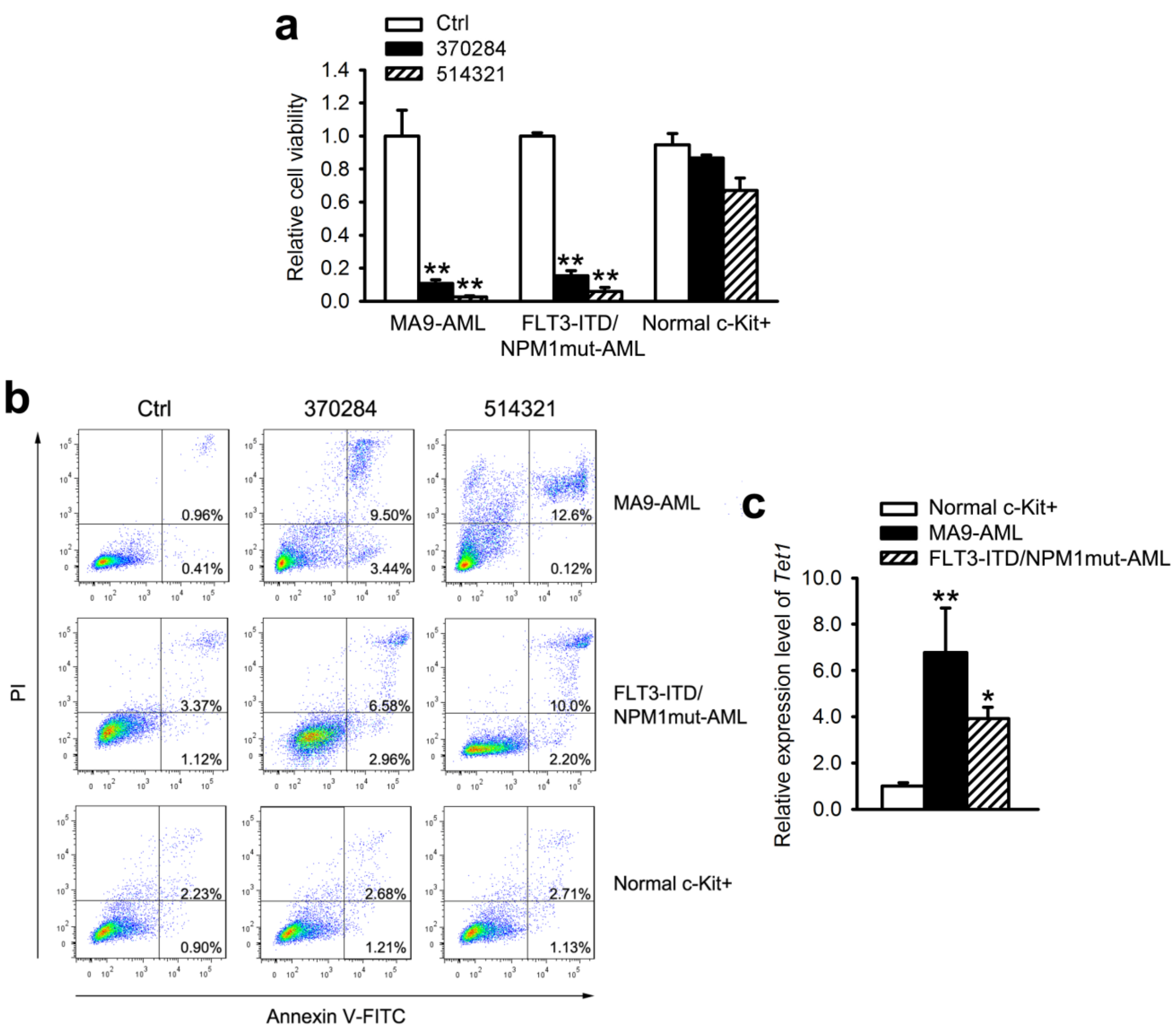

Supplementary Figure 8. Comparison of effects of NSC-370284 and UC-514321 between AML cells and normal hematopoietic stem/progenitor cells (HSPCs; herein, c-Kit$^+$ BM progenitor cells). BM cells of leukemic mice with *MLL-AF9* fusion (MA9-AML) or *FLT3*-ITD/*NPM1*^{mut}, and c-Kit$^+$ HSPCs from a pool of 6 healthy donor mice were cultured in liquid medium. Cells were treated with 50 nM NSC-370284, UC-514321, or DMSO as control. MTS (**a**), apoptosis assays (**b**) and qPCR (**c**) were conducted 3 days after cell seeding or drug treatments. Error bar indicates SD of triplicate experiments.

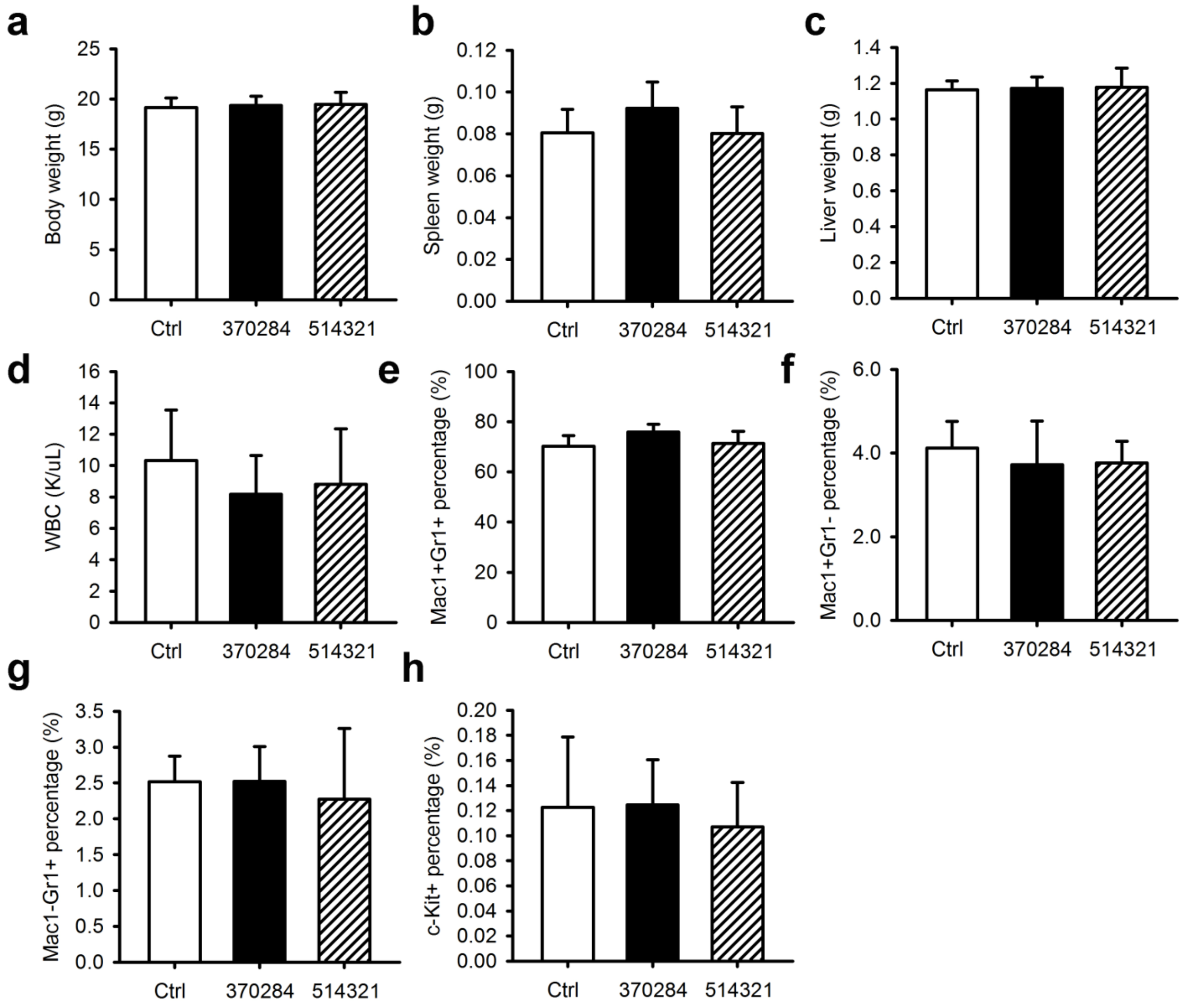

Supplementary Figure 9. Acute toxicity profiling of NSC-370284 and UC-514321. Wild-type mice (*n*=5 for each group) were injected with DMSO (control), 2.5 mg/kg NSC-370284 or UC-514321, *i.p.*, once per day, for 10 days. 24 hrs after the last administration, body weights (**a**), weights of spleens (**b**), livers (**c**), total PB WBC counts (**d**), BM $Mac1^{+}Gr1^{+}$ (**e**), $Mac1^{+}Gr1^{-}$ (**f**), $Mac1^{-}Gr1^{+}$ (**g**), and $c\text{-}Kit^{+}$ (**h**) populations were analyzed.

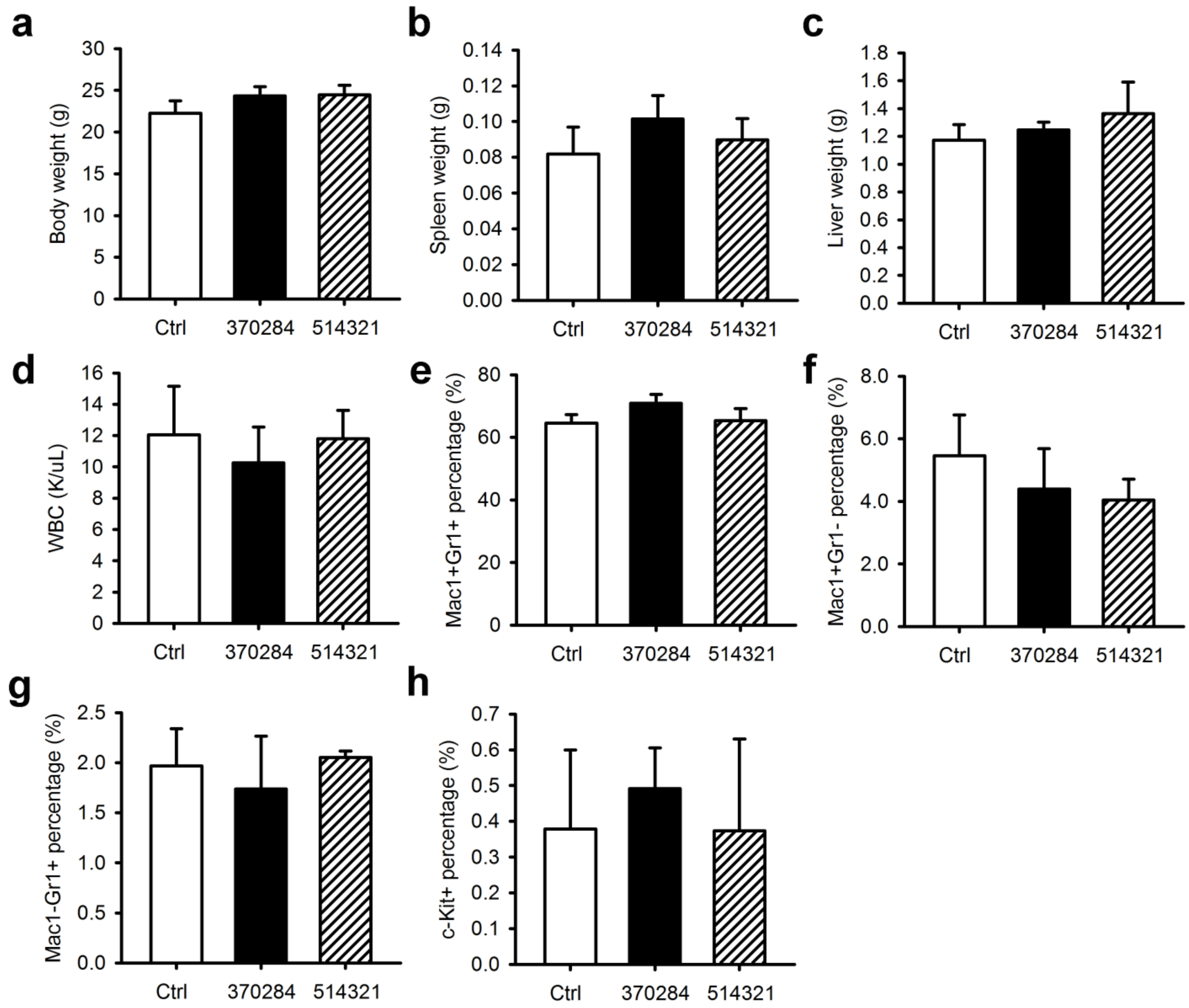

Supplementary Figure 10. Long-term toxicity profiling of NSC-370284 and UC-514321. Wild-type C57BL/6 mice (*n*=5 for each group) were injected with DMSO (control), 2.5 mg/kg NSC-370284 or UC-514321, *i.p.*, once per day, for 10 days. 200 days after the last administration, body weights (**a**), weights of spleens (**b**), livers (**c**), total PB WBC counts (**d**), BM $Mac1^{+}Gr1^{+}$ (**e**), $Mac1^{+}Gr1^{-}$ (**f**), $Mac1^{-}Gr1^{+}$ (**g**), and c-Kit^{+} (**h**) populations were analyzed.

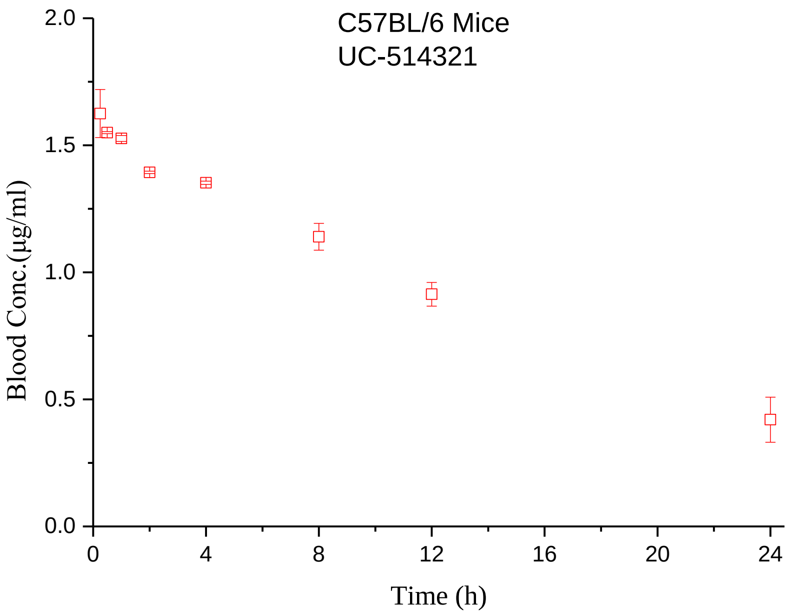

Supplementary Figure 11. Blood concentration of UC-514321 after intraperitoneal injection. Error bar indicates SD of triplicate experiments.

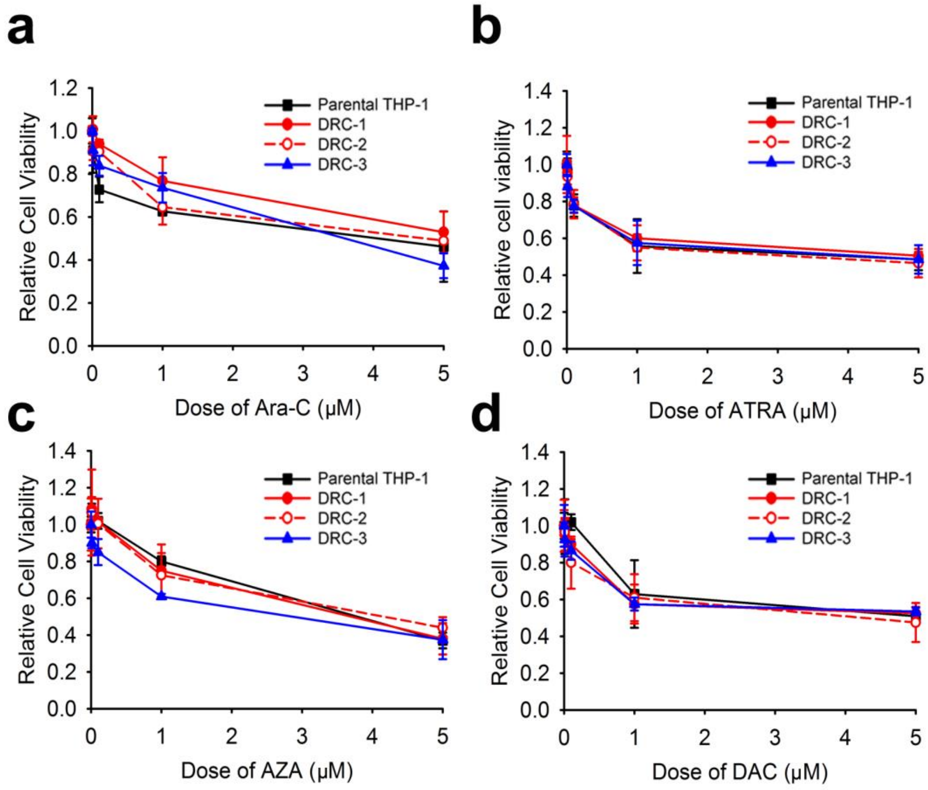

Supplementary Figure 12. Effects of standard chemotherapy reagents on the cell viability of THP-1 NSC-370284-resistant clones. Three representative THP-1 NSC-370284-resistant clones (DRC-1-3) and the parental control were treated with cytarabine (AraC) (**a**), all-trans retinoic acid (ATRA) (**b**), azacytidine (AZA) (**c**) and decitabine (DAC) (**d**) at indicated doses. Cell viability was tested 48 hours after the treatments. Error bar indicates SD of triplicate experiments.

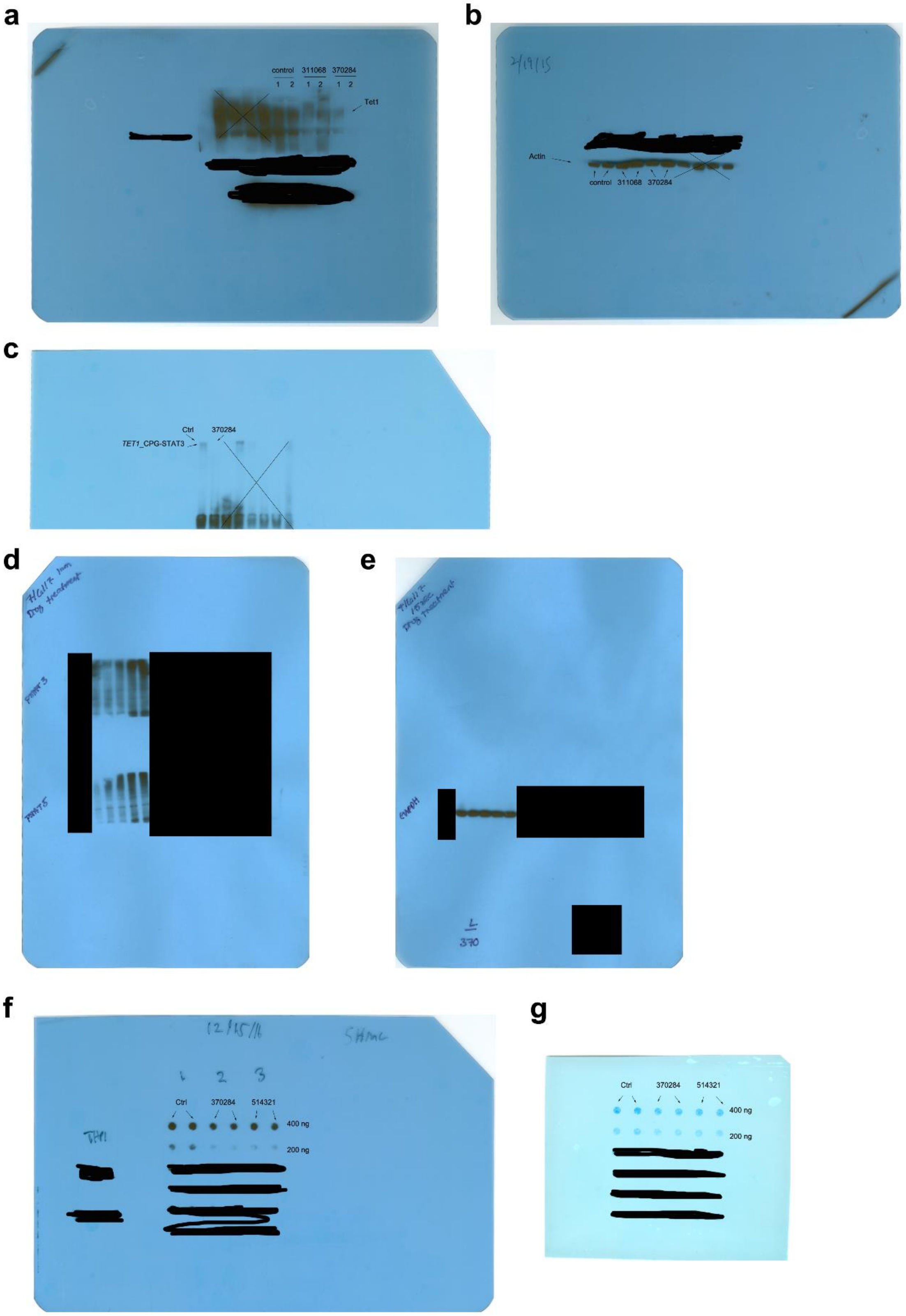

Supplementary Figure 13. Original scans of Western blotting and dot blotting results.

SUPPLEMENTARY REFERENCES

1. Fatrai, S., Wierenga, A.T., Daenen, S.M., Vellenga, E. & Schuringa, J.J. Identification of HIF2alpha as an important STAT5 target gene in human hematopoietic stem cells. *Blood* **117**, 3320-3330 (2011).
2. Nagy, Z.S., *et al.* Genome wide mapping reveals PDE4B as an IL-2 induced STAT5 target gene in activated human PBMCs and lymphoid cancer cells. *PLoS One* **8**, e57326 (2013).
3. Rani, A., Greenlaw, R., Runglall, M., Jurcevic, S. & John, S. FRA2 is a STAT5 target gene regulated by IL-2 in human CD4 T cells. *PLoS One* **9**, e90370 (2014).
